# Curriculum Mapping for Curriculum Development: The Notion of “Curriculum Barcoding” in View of the Saudi Medical Education Directives Framework (SaudiMEDs)

**DOI:** 10.7759/cureus.29886

**Published:** 2022-10-03

**Authors:** Badr A Alsayed, Ahmad A Omer

**Affiliations:** 1 Internal Medicine, University of Tabuk, Tabuk, SAU; 2 Surgery, Prince Sattam Bin Abdulaziz University, Alkharj, SAU

**Keywords:** saudi arabia, saudimeds, curriculum mapping, curriculum development and evaluation, curriculum barcoding, competency-based medical education

## Abstract

Objectives

This study aims to map the curriculum of the Faculty of Medicine at Tabuk University to assess its comparability with the SaudiMEDs competency framework.

Methodology

We developed a checklist based on the essential clinical presentations and skills listed in the SaudiMEDs to map our curriculum and determine the comparability. This cross-sectional descriptive study started on 1 September 2015 until 29 February 2016. The coordinators of the 34 modules completed the checklist and identified whether each clinical presentation or skill is taught in their relevant modules.

Results

Results showed that our curriculum is lacking in 3.9% of the clinical presentations and 23.9% of the skills deemed necessary by the SaudiMEDs, and require attention. Deficient skills were mainly hospital-based ones. The project yielded a content “expertise” map regarding where the main domains of knowledge and skills in the SaudiMEDs framework are addressed in our curriculum. The “SaudiMEDs barcode” is generated that we hypothesize as a novel method for the description of our program in relation to the national competency framework.

Conclusion

Curriculum mapping is a powerful tool for curriculum improvement. Our study elucidated a minor gap in the knowledge domains but a significant one in the essential skills in relation to the SaudiMEDs. We recommend structured training during the internship period as an essential supplement to undergraduate medical qualifications. During our experimentation with curriculum mapping, we articulated the “SaudiMEDs barcode” that we suggest as a novel method for curriculum alignment to the matrix of national competency and, hopefully, to aid in the accreditation projects.

## Introduction

The history of medical education has attracted substantial interest over the past few years, and medical education has moved toward outcome-based education (OBE) [1-10]. This paradigm has been necessitated by increasing concerns about accountability, cost-effectiveness, and rising expectations of the community regarding what future physicians can do to meet their needs [1,3,5,11-14]. Enthusiasm for competency-based medical education (CBME) has translated into the establishment of competency-based frameworks for medical graduates in many countries, for example, the Can Meds (Competency framework approved by the Royal College of Physicians and Surgeons of Canada); Tomorrow’s Doctor; the Scottish Doctor; and the Accreditation Council for Graduate Medical Education (ACGME) [4,8,15-17]. Moreover, many medical schools are adopting these frameworks in their curricula [6,18]. In contrast to the traditional time-based model, CBME de-emphasizes time and concentrates on how the student can actually perform at the end of their course of study [2,4,10,11,18]. Despite the wide reputation and implementation of this educational strategy, some challenges remain to be addressed, which include the definition of competencies and the development of authentic methods of assessment of these competencies [5]. In addition, the lack of shared discourse and terminology is another reported limitation that currently compromises the full promise of CBME [4,12,13,17,19].

CBME and evolving national competency frameworks in Saudi Arabia

Saudi Arabia was not uncompetitive and has made recognizable efforts recently to establish national competency frameworks for medical graduates [10,16,20-22]. Such national frameworks aim to provide a common platform on which the curricula of medical schools in the kingdom can be grounded and against which all Saudi medical graduates can be compared in terms of skills and knowledge [10,21,23]. This approach has been deemed necessary, particularly in view of the unprecedented expansion in the establishment of medical colleges in the kingdom within the past decade [15,21,23]. In addition, the development of such national frameworks would bring medical education in line with the values, traditions, and cultural background relevant to Saudi Arabia [6,15,16,24]. These efforts have been supported by the birth of the National Commission for Academic Accreditation and Assessment (NCAAA) and the Saudi Medical Education Directives (SaudiMEDs) competency frameworks [22]. The NCAAA, founded in 2004 and responsible for the accreditation and quality assurance of postsecondary training programs, drafted the “Learning Outcomes for bachelor's degree programs in Medicine” in 2010, which comprises five knowledge and skill domains [10,16,22]. Later, and with regard to medical education, an initiative led by a committee of deans of medical colleges in Saudi Arabia, produced an initial draft of the SaudiMEDs in 2010, which was later updated in 2015. The NCAAA and the SaudiMEDs frameworks were cross-referenced to ensure congruence and consistency between the two national frameworks. All medical schools in Saudi Arabia are now required, as part of their accreditation process, to demonstrate that their curricula include the competencies and domains of knowledge and skills that are in accordance with these national frameworks [22].

Curriculum mapping for program development

Harden defines curriculum mapping as “the process of indexing elements and linking them, as well as incorporating other phenomena such as people and timetable” [7]. A curriculum map can be assembled by examining a curriculum through different perspectives, including learning outcomes, subject areas, teaching and learning opportunities, and assessment strategies [25,26]. It has been asserted that the functional linkages between the different objects in a map provide more information than would otherwise be the case with a linear representation of these objects [19,25]. Such a graphical representation of the contents of a curriculum depicts what is taught and elucidates how and where topics are covered, thus making the curriculum more transparent and easily accessible to all concerned parties in the learning process [19]. Curriculum mapping is therefore considered to be a powerful tool for curriculum development because it can highlight gaps as well as redundancies in the formal curriculum [5,9,12,19,27,28]. Lately, several medical training programs and institutions in Saudi Arabia have attempted to map their curricula to certain international and national competency frameworks in response to the growing concerns over improving the quality of their programs and graduates and to meet the requirements of accreditation and quality assurance processes [2,6,14,26].

The SaudiMEDs competency framework

To establish the background for this study, this section provides more details regarding the national competency framework for medical graduates in Saudi Arabia: the SaudiMEDs. The project was launched in 2009 and was planned to be carried out in three phases. The first and second phases have been concluded, while the third phase is still ongoing [16,22]. Phase 3 aims to provide detailed specifications of the competencies expected at the end of the internship year, which, in this initiative, is considered to be an integral part of the basic medical curriculum. The SaudiMEDs comprises six main domains, 17 essential competencies, and 80 learning outcomes that should be fulfilled by all undergraduate medical programs in the kingdom [21,22]. The framework also consists of 178 clinical presentations, classified according to body systems, and 123 essential skills related to six categories, which all medical graduates should learn by the time of their graduation [29]. The SaudiMEDs framework aims to specify the core abilities of the “future Saudi doctor” and serves as a mechanism to ensure the comparability and equivalence of the outcomes of undergraduate medical education all over the country. The framework is meant to inform undergraduate medical curricula in Saudi Arabia and not to replace them, thereby preserving medical schools’ autonomy in the delivery of high-quality medical education that is still based on the schools’ particular contexts and individual needs [16,21,22].

Curriculum overview of the medical college

The Faculty of Medicine at the University of Tabuk implements an outcome-based, community-oriented curriculum designed as a modular system and has three phases that are delivered over six years. The internship period, the seventh year, is not yet a part of our undergraduate curriculum. Initially, the curriculum was adopted from the medical college at King Abdul-Aziz University as a “mother college,” and it embraces a multilayered, longitudinally, horizontally integrated, and interdisciplinary approach [4,17]. Each module is organized based on a set of learning outcomes and appropriate teaching, learning, and assessment strategies to help students achieve those outcomes. Phase 1 is the preparatory year, which involves general science subjects and the scientific English language. Phase 2 represents the second and third years and comprises system-based, integrated modules which deliver basic sciences and applied clinical knowledge. In the third and final phase, students receive comprehensive discipline-based clinical studies and rotations. Successful performance in each phase is mandatory for the student to advance to the next phase.

Study rationale and objectives

Curriculum development in our college was a major concern for successive administration leaders [16,23,27]. In 2011, in collaboration with the University of Queensland in Australia, an initial evaluation of the curriculum was conducted with regard to its comparability with the Liaison Committee on Medical Education (LCME) and the United States Medical Licensing Examination (USMLE) standards. Recently, with the release of the NCAAA and SaudiMEDs competency specification reports, it became mandatory for all Saudi medical colleges to demonstrate the alignment of their curricula with these national frameworks as part of accreditation and recognition of their programs in the kingdom [9,16,21].

This study aims to assess the degree of congruence between our curriculum and the SaudiMEDs, and hence the NCAAA system because the two were cross-referenced successfully, as mentioned in the foregoing [9,16,17,27,28]. We aim to map our curriculum to determine the extent to which it is aligned with the SaudiMEDs and to identify any gaps in respect of accreditation of our program and its future development [9,12,19,27].

We believe that this exercise has fundamental advantages and implications for our college. In addition to fulfilling the requirements of the accreditation process [6,19,27], which has already been launched, it may delineate areas of deficiency and redundancy in our curriculum and enable curriculum planners to tailor these in a more efficient manner [6,19,22,26-28]. This is particularly vital in view of the tight schedules that characterize modular systems such as the one our college employs [28]. Moreover, the curriculum map may improve transparency and access for all stakeholders, for example, students, teachers, module coordinators, and program leaders, to the curriculum and help them better plan their learning, teaching, and development actions, with all working toward a common goal [5-9,11,15,20,26,27]. This study may provide evidence of curriculum evaluation and development approaches in our college, which are essential for internal quality assurance processes, and reassure different stakeholders regarding the feasibility and outcomes of our program [2,6,14,26]. Finally, yet importantly, it may encourage other medical schools to perform the same exercise, which could eventually provide crucial feedback to the steering committee of the SaudiMEDs initiative.

## Materials and methods

This study was approved and funded by the Deanship of Scientific Research at the University of Tabuk (UT-127-21-2020). This is a descriptive-analytical study that aims to map the curriculum of the Medical College of the University of Tabuk against the SaudiMEDs specification framework. We developed a checklist to collect data from all modules based on the list of clinical presentations and skills that are deemed essential to be covered and mastered by students upon graduation as outlined in the SaudiMEDs framework [29]. The checklist consisted of the 178 clinical presentations and the 123 essential skills listed in the SaudiMEDs specification document. The checklist specifies where each clinical presentation or skill is taught in our modules, how much time is spent on that task [6,19], and how is it assessed, postulating that the coverage of these clinical topics and skills would indicate that our curriculum is congruent with the SaudiMEDs framework because competency is considered to be an aggregation of “knowledge and ability” [5,6,8,15,18,19,26]. The aim was to develop a content or “expertise” map that breaks down the subjects taught in our modules and reallocates them to form clusters of expertise around the main subject areas contained in the SaudiMEDs [6,14,19,26-28]. We invited four module coordinators randomly to the pilot phase and asked them to complete the checklist and make comments regarding the process and the number of instructions provided. A message was disseminated to the faculty regarding the project aims and its advantages to our college in a formal meeting led by the dean, and the timeframe for the completion of the project was also discussed. Then, each module coordinator, with the help of other instructors who teach the course, was asked to fill out the checklist based on the module’s study guide under supervision and with the assistance of the researchers [8,20].

Following the completion of the checklist by all module coordinators, the researchers revised all the data and checked for deficiencies and/or inconsistencies, and wherever necessary, module coordinators were contacted again to clarify and verify their entries. Researchers also checked to ensure that the entries in the checklist were based on what is documented in the study guide of the relevant module so as to reflect the situation in reality [14,26]. In the final stage, we gathered the information from all the checklists and combined it all into a final, single checklist of the same kind. Data analysis was first done manually. In the final checklist we calculated the percentages of the essential clinical presentations and skills not covered in the program. Then we used the Excel software (Microsoft Inc, USA, 2010) to draw graphical representations of where those topics and skills are addressed in our taught modules and academic years. The aim was to draw a content map of the curriculum oriented around the main knowledge and skill domains of the SaudiMEDs competency framework. Also, we mapped the clinical presentations and essential skills contained in the SaudiMEDs against the taught modules in our program using Excel (Microsoft Inc, USA, 2010) to provide further evidence of how and where the formers are addressed in our curriculum.

## Results

**Figure 1:**
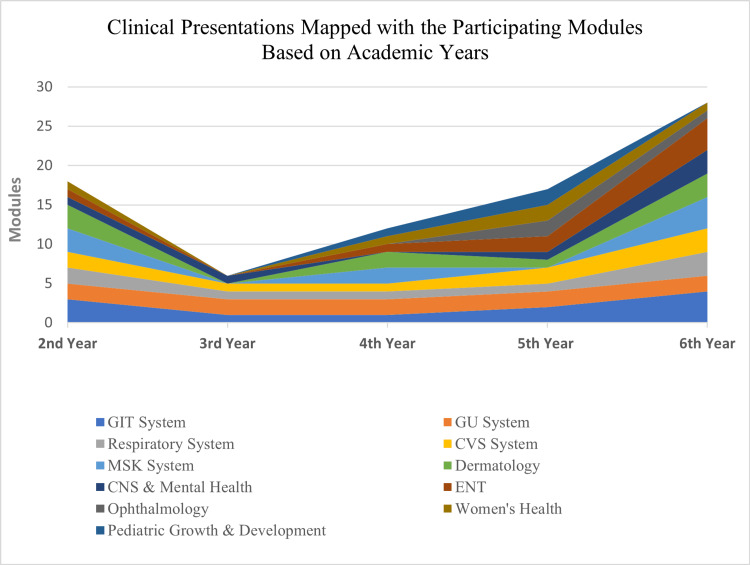
The clinical presentations mapped with the participating modules—based on the academic year in which they were taught—grouped according to the main subject areas of the SaudiMEDs framework.

**Figure 2:**
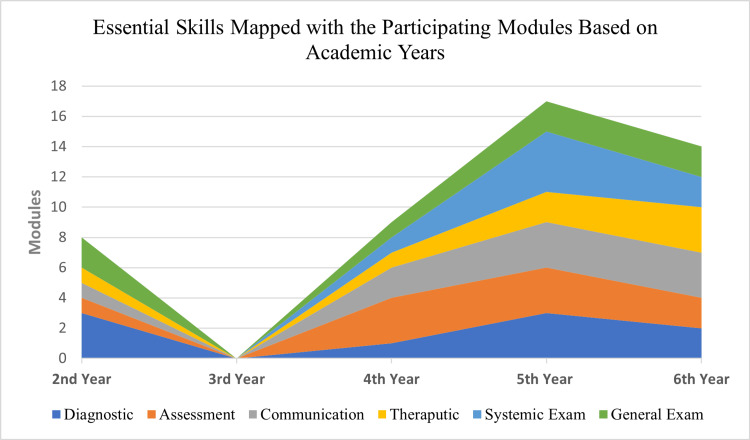
The essential skills mapped with the participating modules are based on academic years oriented around the main skill domains of the SaudiMEDs framework.

**Figure 3:**
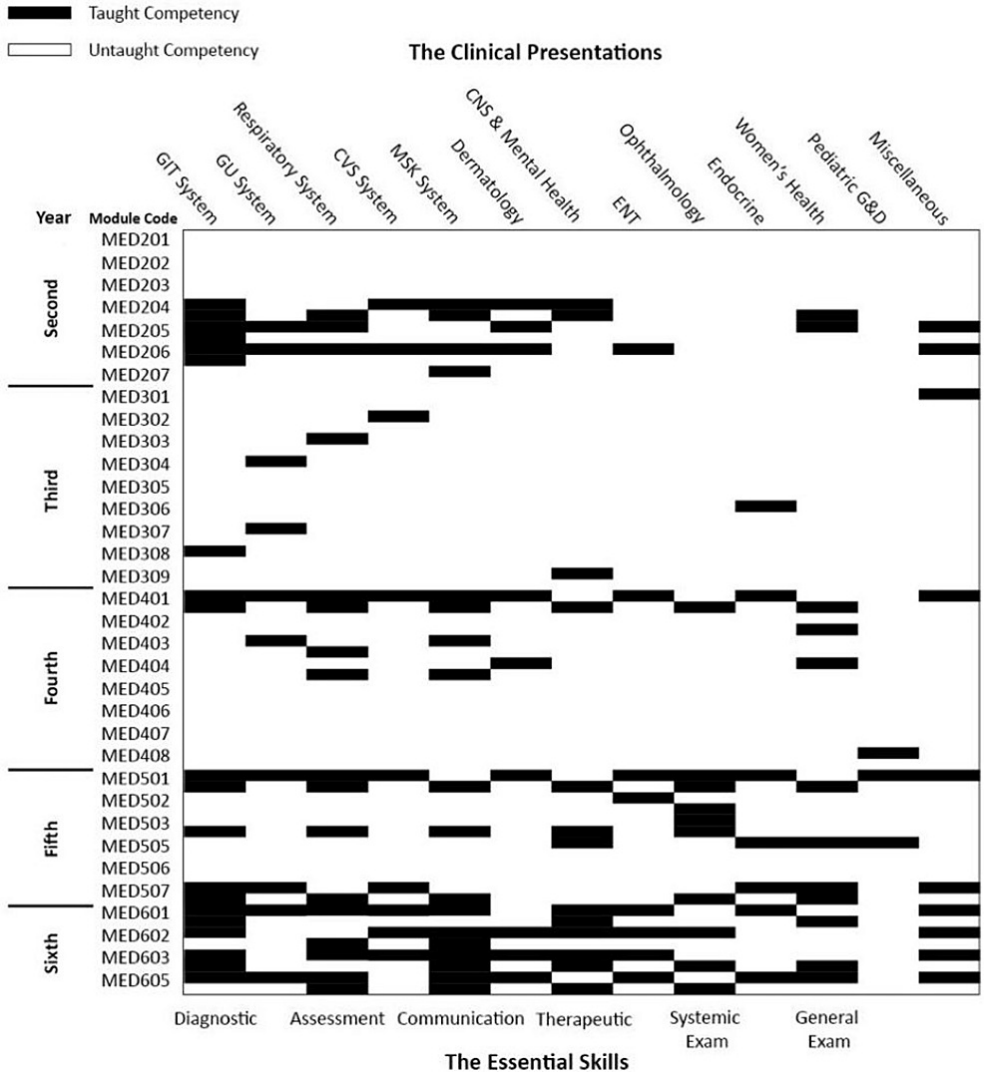
The SaudiMEDs barcode of the curriculum of the faculty of medicine at Tabuk University. Each taught module comprises two rows: an upper one representing clinical presentations and a lower one counting for the essential skills white cells indicate untaught competency.

**Figure 4:**
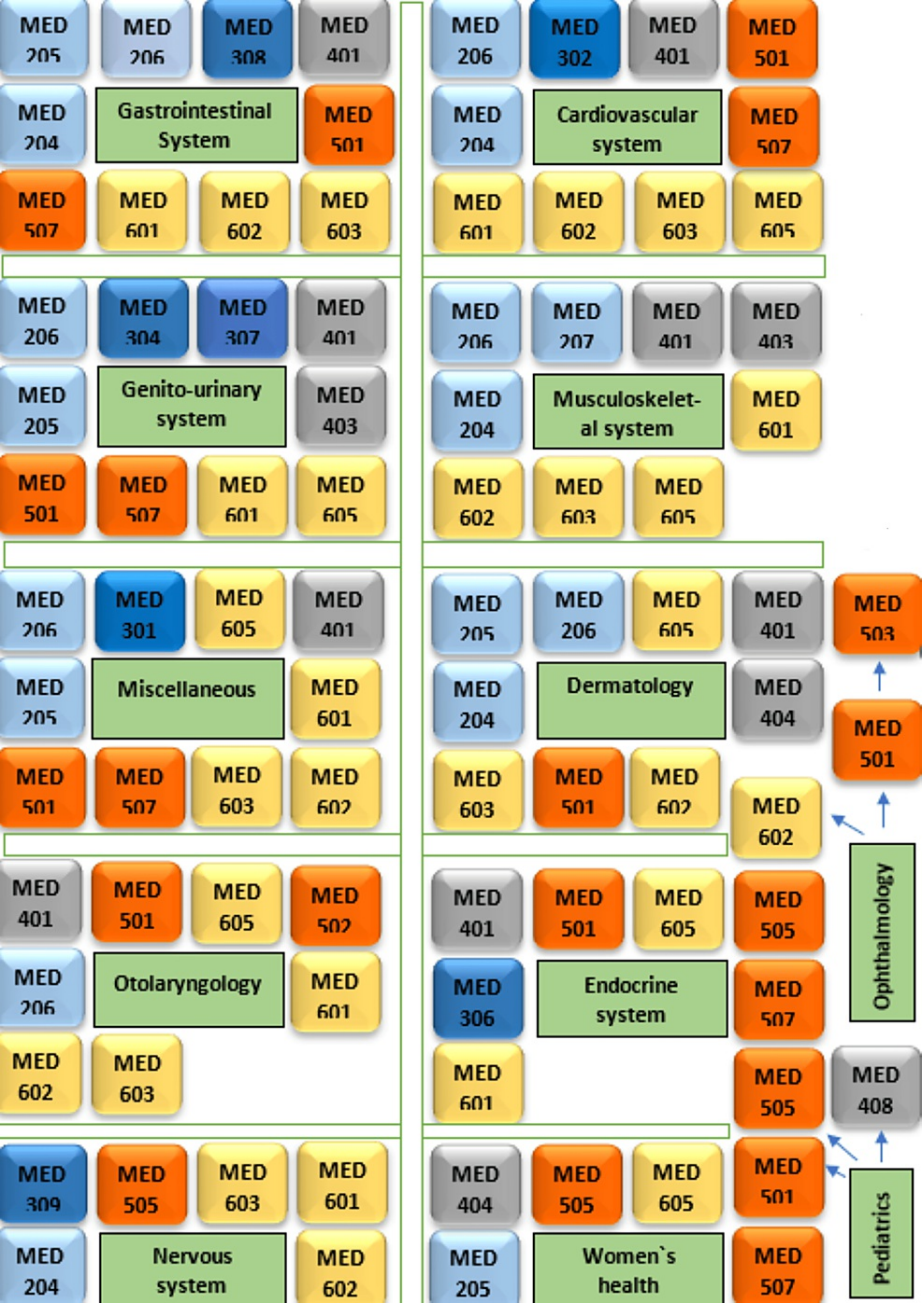
The clinical presentations map. MEDxxx = Module's Code. The first number (on the left) corresponds to the academic year. Color Code: Yellow: Sixth Year, Red: Fifth Year, Gray: Fourth Year, Dark Blue: Third Year, Light blue: Second Year.

**Figure 5:**
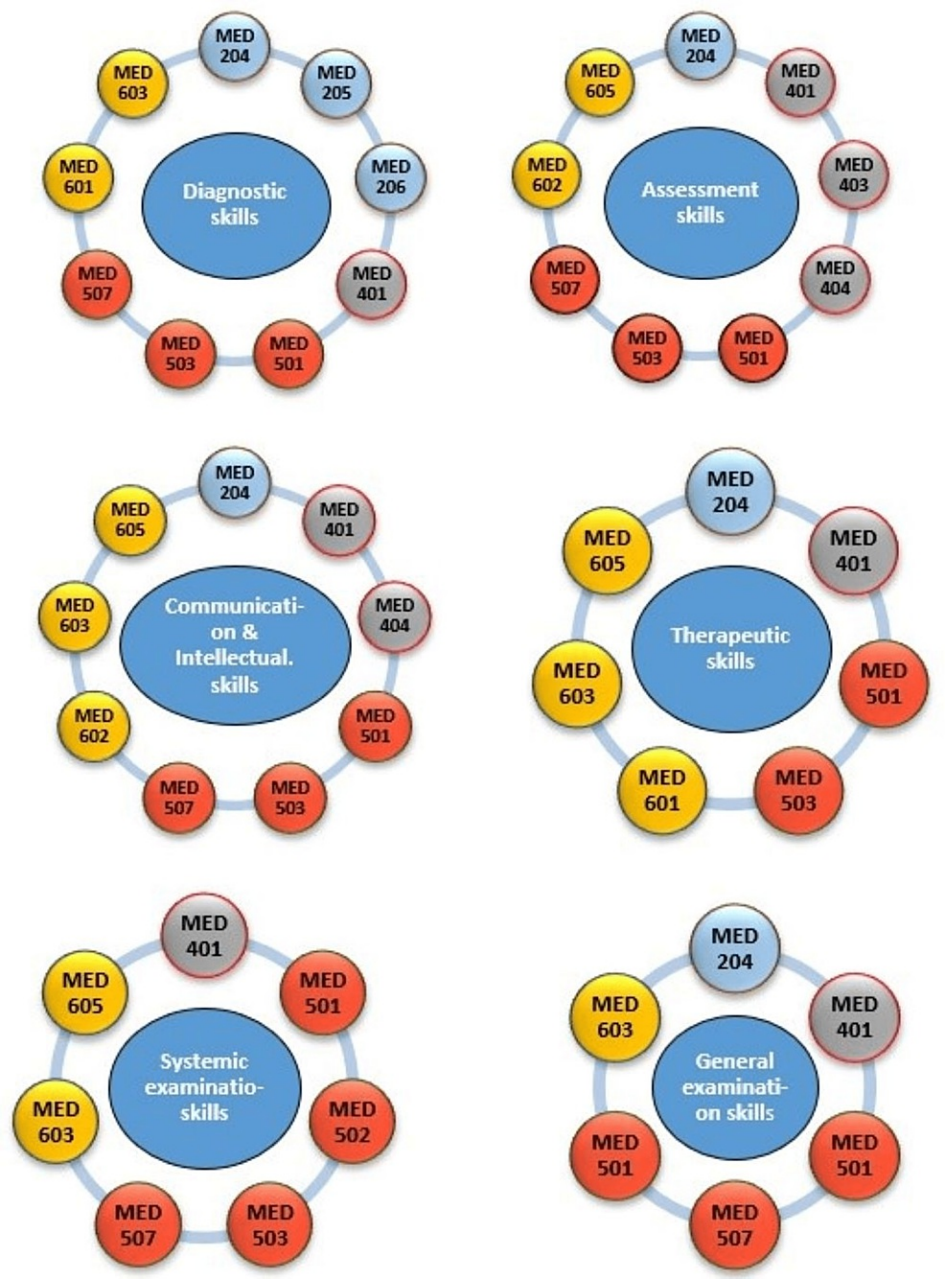
Snapshot from the essential skills maps with the modules oriented around the main skill domains of the SaudiMEDs framework. MEDxxx = Module's code. The first number (on the left) corresponds to the academic year. Color code: Yellow: Sixth Year, Red: Fifth Year, Grey: Fourth Year, Blue: Second Year.

Twenty-seven module coordinators completed the checklist for each module reporting in total the 34 modules taught in the College. The clinical presentation map shows the contribution of each academic year to the main subject areas of the SaudiMEDs (Figure 1). The coverage of these clinical presentations in our curriculum is approximately 96.1%. Figure 2 shows the skills map, which highlights the contribution of each academic year to the achievement of the main skill domains as indicated in the SaudiMEDs framework. It was found that our curriculum-excluding the preparatory and internship years-currently contains around 76.1% of the skills deemed to be necessary by the SaudiMEDs. The color mix in each column of the academic years reflects horizontal integration, whereas the continuity of each color through all academic years represents vertical integration. Plotting the taught modules against the main skill and knowledge domains of the SaudiMEDs specification matrix in Excel (Microsoft Inc, USA, 2010) produced a characteristic shape of “barcode”, which identifies our program in comparison to the national competency framework, as shown in Figure 3. Also, we drew visual maps which show the taught modules clustered around the main knowledge and skill domains of the SaudiMEDs framework to demonstrate the contribution of the formers to the achievement of the latter (Figures 4, 5), respectively.

## Discussion

Curriculum mapping is a process wherein the curriculum is analyzed based on its basic units, and links are made to show how these units are related to each other [6]. This study aims to create a map in which the contents of our curriculum that is taught are clustered around the main subject and skill domains of the SaudiMEDs competency framework.

Curriculum mapping

The clinical presentations and skills map produced by our study provide a picture of where all of these areas of knowledge and skills are taught and yield a graphical orientation of the different academic years of our program through plotting the SaudiMEDs domains. This visual representation of the relationship between the modules in each academic year and their contribution to the SaudiMEDs areas of expertise may provide students and faculty with a picture of the overall organization of the curriculum and help them to prepare for their teaching, learning, and assessment activities [19,25]. The map may also set the stage for legitimate curricular management decisions to reorganize the distribution of subjects and skills across the modules and redefine the assessment strategies more appropriately. We have identified several clinical presentations and essential skills that our curriculum is deficient in, in relation to the SaudiMEDs, and therefore need to be addressed. However, and understandably, most of the uncovered essential skills are hospital-based and hence would more likely be taught and mastered in the internship period. This emphasizes the importance of setting a structured program for the internship period as part of the undergraduate training, which is already proposed in the third phase of the SaudiMEDs project. Also, some redundancies have emerged and may require attention in the future plans for curriculum development. For example, dermatology and otolaryngology topics appeared to be dealt with in a relatively large number of modules, and if necessary, revision of these subjects may result in some additional space to bridge the gap with regard to the deficiencies. The ability to delineate such gaps and redundancies in the curriculum is one of the prime advantages of curriculum maps, as indicated in the literature [6,19,22,26-28].

Furthermore, the map provides insights into the degree of integration in our program, which is another one of its advantages because the curriculum involves a horizontal and vertical integration approach, particularly in the preclinical phase. Thus, at a glance, the map demonstrates the level and the modules that contribute to each subject or skill area. Thereafter, decisions regarding the appropriateness of the stage and the courses in which each subject or skill is taught could be effected. While this exercise may provide a model to the growing community of medical schools in Saudi Arabia wishing to cross-reference their curricula against the SaudiMEDs framework, it may also demonstrate to the wider community of medical educators that curriculum mapping remains an essential tool for curriculum development.

Curriculum barcoding

Our experimentation with curriculum mapping has yielded a unique barcode-like shape that depicts the relationship between the formal curriculum at our college and the SaudiMEDs framework. The barcode exhibits the alignment of our taught modules in relation to the main knowledge and skill domains of the national framework. This approach has sparked in our minds the notion of “curriculum barcoding”. Thinking loudly, we asked ourselves, is it possible to “barcode” the curriculum in view of a national standard? Would it be a legitimate mechanism to define the curriculum in relation to the minimum training requirements stipulated by the SaudiMEDs? Would it help the wider community of medical schools in the kingdom to define their curricula in view of the national specifications and requirements? Obviously, it might be too early to answer those questions. However, the idea might be appealing given the increased aptitude for standardization and homogenization of medical education and training in the present era of globalization [30]. In addition, the notion of barcoding might be conducive to the growing enthusiasm for the adoption of national and international competency frameworks currently noted in different jurisdictions. It might be possible to translate the basic medical program into a barcode in terms of where the essential matrix of knowledge, skills, and competencies described by the SaudiMEDs are addressed in the curriculum. Once that is done, the “SaudiMEDs barcode” might be used to codify and test the alignment of the basic medical curriculum to the requirements of the national framework similar to how barcode machines in the market translate an aggregation of columns into the price. The such process might also be endeared in a world dominated by computerization and digital technologies. Of course, the SaudiMEDs are not intended as a formal curriculum; it preserves the autonomy of medical schools to meet the requirements of their local communities. As such, standardization of basic medical programs is not required to allow for barcoding their curricula to the SaudiMEDs. Instead, the process is more likely codifying the curriculum against the minimum requirements set by the national standard, and can therefore be done regardless of the curriculum design and structure.

Once created, the unique shape of the “SaudiMEDs barcode” of a given basic medical curriculum in the kingdom might serve as a novel method to display its “identity” in view of the SaudiMEDs and to demonstrate the congruence between that curriculum and the national standard. Besides, we believe that the barcode might constitute a platform or common language to facilitate communication of curriculum content and characteristics in relation to achievement of the national requirements among the community of medical education in Saudi Arabia. We believe that the “SaudiMEDs barcode” of a given college might help the responsible authorities to figure out the degree of alignment between the college's curriculum and the national specification requirements. As such, the process might also have implications to the overseeing and accreditation projects of basic medical programs in the country. The above-mentioned advantages might be few examples of others if the “SaudiMEDs barcode” turned to be applicable. However, and understandably, this quantitative approach alone might not be enough for curriculum assessment and qualitative and other evaluative methods in this concern might be essential. We are aware that this novel idea is still in its infancy and therefore requires further elaboration and testing to endorse its applicability and usefulness in our setting and perhaps other medical schools in the kingdom. This should be the subject of future studies. Lastly, we call for initiation of immense discussion among the community of medical schools in the kingdom to explore the utility and the pros and cons of the “SaudiMEDs barcode” as a promising method of curriculum description and evaluation with regard to the set of national requirements.

## Conclusions

Curriculum mapping is a powerful tool for curriculum development and improvement. The curriculum in our medical college is comparable to and includes most of the skills and knowledge domains of the SaudiMEDs competency framework; however, some gaps need to be addressed. The curriculum map provided a tool for the recognition of unnecessary overlaps in the program and an insight into the appropriateness of the integrated teaching approaches employed in the college. We hypothesized a novel method to test the alignment of our curriculum, and perhaps other corresponding programs in the kingdom, to the SaudiMEDs framework. We described the former as the “SaudiMEDs barcode”, which we hope to have implications to fulfillment of all the desired competencies by the future Saudi doctors and to promote overseeing and accreditation of basic medical programs in the kingdom. Data generated from this exercise are useful for informing the decisions and actions that might be taken to improve the curriculum. In addition, it emphasizes the need for a structured training system in the internship period as part of the undergraduate medical program. Further studies in this field are recommended in our college to help in providing continuous assessment and optimization of our curriculum. At the national level, we recommend further studies on curriculum mapping with established guidelines to promote transparency, accessibility to, and improvement of basic medical curricula in the kingdom. Besides, we anticipate further work to test the utility of the “SaudiMEDs barcode” as a promising tool to ensure comparability and alignment of the basic medical programs in Saudi Arabia to the national training standards.
